# Radiographic Enhancing Progression Without Molecular or Histologic Progression in IDH‐Mutant Grade 2 Astrocytoma Treated With an IDH Inhibitor: A Case Report

**DOI:** 10.1155/crnm/5250404

**Published:** 2026-07-30

**Authors:** Carolina Pusec, Karam Han, Krithi Gopinath, Michelle Mackay, Alan Lozier, Tejus A. Bale, Ankush Bhatia

**Affiliations:** ^1^ Department of Neurology, University of Wisconsin Madison, Madison, Wisconsin, USA; ^2^ Department of Pathology and Laboratory Medicine, University of Wisconsin Madison, Madison, Wisconsin, USA, lhsc.on.ca; ^3^ Department of Neurology, University of Wisconsin School of Medicine and Public Health, Madison, Wisconsin, USA, uwm.edu; ^4^ SSM Health Neurosciences, SSM Health St. Mary’s Hospital, Madison, Wisconsin, USA, ssmhealth.com; ^5^ Department of Pathology and Laboratory Medicine, Memorial Sloan Kettering Cancer Center, New York, New York, USA, mskcc.org

## Abstract

Central nervous system (CNS) World Health Organization (WHO) Grade 2 isocitrate dehydrogenase (IDH)‐mutant astrocytomas are classified as low‐grade gliomas with typically slow growth for many years followed by eventual malignant transformation and death. Here, we present a case of a patient with an IDH‐mutant Grade 2 astrocytoma who developed early radiographic tumor progression per Response Assessment in Neuro‐Oncology (RANO) 2.0 as evidenced by new enhancement on MRI T1 postcontrast 4 months after initiation of an IDH inhibitor. Subsequent re‐resection, one month later (or five months postinitiation of ivosidenib treatment), of the enhancing signal retained the Grade 2 designation without any histological or molecular progression per CNS WHO 2021 criteria. Specifically, the Ki‐67 proliferative index remained stable at approximately 2%, and copy number alterations were largely unchanged, including no *CDKN2A/B* homozygous deletion and relatively low chromosome copy number complexity. On histological examination, there was no elevation in mitotic activity and no features of necrosis or microvascular proliferation to suggest a more aggressive phenotype. The tumor demonstrated increased gemistocytic morphology and rare foci of foamy macrophages, as compared to prior. Following the second resection, the patient received sequential radiation therapy and adjuvant temozolomide chemotherapy treatment and has remained clinically stable on observation with surveillance MRIs for over 18 months. This case highlights the importance of re‐resection in IDH‐mutant gliomas, identifies the potential limitations of the response assessment criteria to differentiate treatment effect from true tumor progression, and emphasizes the need for ongoing investigation into novel genetic alterations, molecular signatures, and histologic markers to more accurately predict radiographic tumor behavior and guide more personalized, targeted treatment strategies for patients with IDH‐mutant gliomas.

## 1. Introduction

Gliomas are the most common primary brain tumors, accounting for the majority of malignant central nervous system (CNS) neoplasms [1]. The updated 2021 World Health Organization (WHO) classification of tumors of the CNS has split the classification of adult‐type diffuse gliomas into isocitrate dehydrogenase (IDH)–mutant 1p/19q intact astrocytoma, IDH‐mutant 1p/19q‐co‐deleted oligodendroglioma, and IDH‐wildtype glioblastoma [2]. Grade 2 IDH‐mutant astrocytomas, though classified as low‐grade, are notable for their generally slow growth over several years with eventual malignant transformation and relentless growth leading to neurological morbidity and death [3]. Grade 2 IDH‐mutant astrocytomas are characterized histologically by well‐differentiated tumor cells and relatively low cellularity; the Ki‐67 proliferation index is usually <  4%, mitotic figures are rare, and microvascular proliferation and necrosis are absent [4, 5]. Homozygous deletion of the *CDKN2A/CDKN2B* tumor suppressor gene denotes CNS WHO Grade 4 tumors and is a hallmark of malignant progression with significant prognostic implications [2]. A higher grade tumor (Grade 3 or 4) may be suggested by neuroimaging when contrast enhancement is present [6]. The standard of care (SOC) for Grade 2 IDH‐mutant astrocytomas is maximal safe resection to remove as much tumor as possible while preserving neurological function. The histology and molecular analysis of the resected tissue help guide further treatment, which could involve observation or treatment with radiation and adjuvant chemotherapy with temozolomide versus novel IDH inhibitors [7–10].

Mutations in *IDH1* and *IDH2* are early drivers of gliomagenesis, leading to the production of the oncometabolite 2‐hydroxyglutarate (2‐HG). Elevated 2‐HG contributes to tumorigenesis by altering cellular metabolism and epigenetic regulation, leading to increased double stranded DNA (dsDNA) breaks, genome‐wide CpG island hypermethylation, decrease in NADP+, and inhibition of alpha‐ketoglutarate–dependent enzymes [11–13].

Understanding the role of IDH1/2 in gliomagenesis has led to the development of targeted therapies. One notable agent, vorasidenib, is a CNS‐penetrant dual inhibitor of mutant *IDH1* and *IDH2* and was FDA‐approved in August 2024 for the treatment of IDH‐mutant Grade 2 astrocytomas and oligodendrogliomas following surgery. Vorasidenib demonstrated significant reductions in 2‐HG production in preclinical models, and in the INDIGO trial, it demonstrated significantly prolonged PFS (median 27.7 months vs. 11.1 months in the placebo group) [10]. Ivosidenib, which is an IDH1‐specific inhibitor, was FDA‐approved in 2018 for refractory IDH1‐mutant AML, incorporated into guideline‐based management, and further made available for off‐label use in recurrent low‐grade gliomas [14, 15]. A Phase 1 trial comparing these two agents administered to patients with IDH1‐mutant low‐grade gliomas found that both drugs were able to achieve greater than 90% reduction in tumor 2‐HG levels, indicating robust IDH enzyme inhibition, despite superior CNS penetration with vorasidenib. Importantly, it suggested that ivosidenib compensates for its lower CNS penetration by having high plasma exposure, which allows for effective targeting of IDH‐mutant gliomas [16].

Real‐world clinical data on the effectiveness of IDH inhibitors treatment for IDH1‐mutant gliomas are still emerging. A retrospective study, which included Grade 3 and 4 tumors, evaluated a cohort of 74 patients treated with ivosidenib monotherapy and reported a median progression‐free survival of 31 months, with overall survival not yet reached. While overall results were encouraging (13% of patients achieved a partial or minor response and 64% had stable disease), approximately 23% still experienced disease progression. Notably, the presence of radiographic contrast enhancement at treatment initiation was associated with lower disease control rates, which is a measure of the proportion of patients who derive clinical benefit through tumor response or disease stabilization [17].

Here, we present a case that exemplifies how radiographic signs of progression can be discordant from underlying histologic and molecular stability in IDH‐mutant Grade 2 astrocytomas, revealing key limitations of response assessment and highlighting the importance of re‐resection and molecular‐pathologic analysis in guiding management.

## 2. Patient Case

**FIGURE 1:**
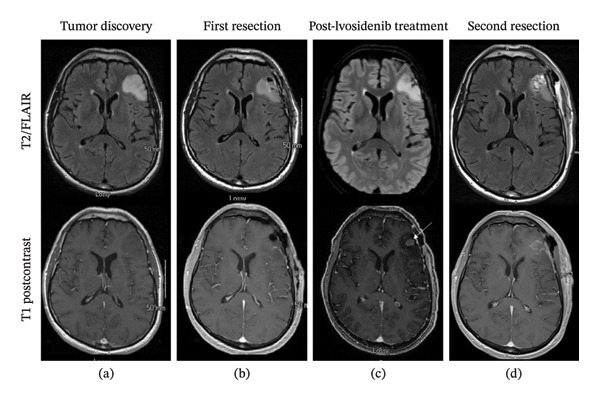
MRI brain imaging. Axial T2/FLAIR and postgadolinium T1‐weighted sequences, depicting the tumor at four time points: (a) initial discovery, (b) following subtotal resection performed 2 months later, (c) after 4 months of ivosidenib treatment with enhancement noted by white arrow in T1 postcontrast imaging, and (d) following second resection 1 month later (5 months after initiation of ivosidenib treatment).

**FIGURE 2:**
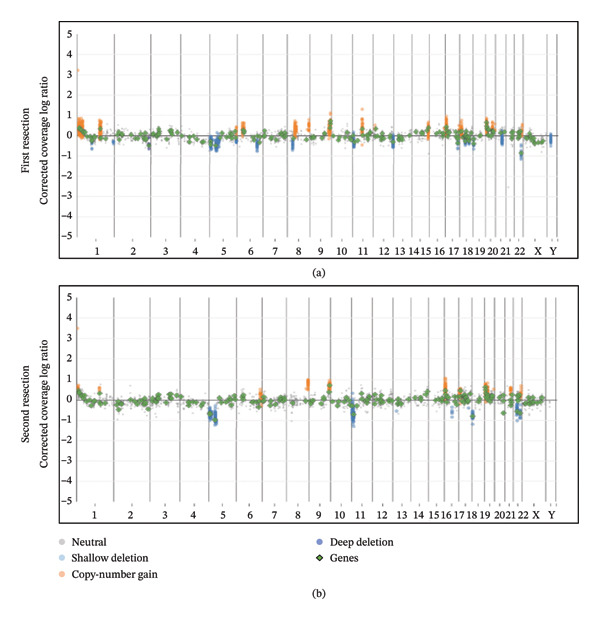
Copy number graph analysis after first and second tumor resections demonstrating chromosomal stability. (a) Copy number profile from the first resection. (b) Copy number profile from the second resection, 5 months postinitiation of ivosidenib treatment. Copy number alterations are plotted as log_2_‐transformed ratios across all chromosomes (*x*‐axis). Copy‐number gains (orange dot), deletions (both shallow and deep are in corresponding shades of blue), neutral regions (gray), and genes (green diamond) are represented.

**FIGURE 3:**
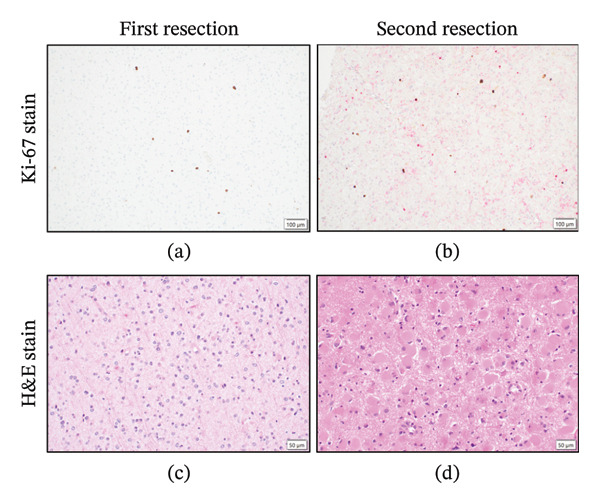
Histopathology following the first and second tumor resections, shown with Ki‐67 and hematoxylin and eosin (H&E) staining. (a) Ki‐67 immunostaining (100 μm field) after the first resection. (b) Ki‐67 immunostaining (100 μm field) after the second resection. (c) H&E staining (50 μm field) after the first resection reveals an infiltrative proliferation of astrocytes with mild nuclear pleomorphism and enlarged, ovoid, hyperchromatic nuclei. Mitotic figures are absent, and there is no evidence of necrosis or microvascular proliferation. (d) H&E staining (50 μm field) after the second resection, 5 months postinitiation of ivosidenib treatment, demonstrates features consistent with gemistocytic differentiation. Tumor cells exhibit large, eosinophilic, glassy cytoplasm with eccentrically placed nuclei, characteristic of gemistocytes. Mitotic activity, necrosis, and microvascular proliferation remain absent.

The patient is a 68‐year‐old otherwise healthy male who was initially evaluated for asymmetric hearing loss of his right ear with tinnitus that progressively worsened over 10 years with chronic headaches. Given this asymmetry, MRI of the brain was recommended which demonstrated an anterior inferior left frontal lobe mass with a focus of cortical and subcortical mass‐like T2/FLAIR hyperintensity with no corresponding enhancement on T1 postcontrast (Figure 1a). The lesion measured approximately 3.9 × 3.2 × 3.1 cm in size. There was no significant mass effect of the left frontal horn, no internal calcification or blood products on gradient echo sequence, or diffusion restriction to suggest ischemia. Additionally, the patient did not have the T2/FLAIR mismatch sign, which is a radiographic sign that has high specificity for IDH‐mutant astrocytomas but not sensitivity. He has never had a seizure and denied having any difficulty with speech or swallowing, nor focal deficits including paresthesia or weakness. About 2 months after tumor discovery, the patient underwent his first subtotal resection (Figure 1b), which was an awake craniotomy with no complications and was on Keppra for 2 weeks. During the awake craniotomy, the patient had subtle right arm weakness; therefore, additional resection was not pursued. Next‐generation sequencing (NGS, Tempus) demonstrated the following mutations: *IDH1 (p.R132H), TP53 (p.I254N),* and *ATRX (p.K955*). There was no *CDKN2A/B* homozygous deletion or *1p/19q* codeletion. Furthermore, there was no significant chromosome copy number complexity, and copy number plots showed chromosomal stability based on the observed copy‐number alterations consisting primarily of low‐level gains and deletions, without high‐level amplifications or broad chromosomal changes (Figure 2a). The tumor was also microsatellite stable and had a low tumor mutational burden (2.1 mutations/Mb). Lastly, there was positive detection of *MGMT* methylation via methylation‐specific PCR technology with a methylation score of 34.83 (threshold ≥ 3.4 defined as positive), and this was via an *MGMT* methylation assay. Based on IHC staining, tumor cells were positive for mutant *IDH1* (p.R132H), p53 mutation (*TP53* missense), loss of expression of *ATRX* (stop gain) and *OLIG2*, and the Ki67 labeling index was low (less than 5%; Figure 3a). Histological findings from biopsy which contained cortex and white matter showed wide infiltration by a low cellularity tumor with morphological features characteristic of a low‐grade astrocytoma. The tumor cells were round to slightly ovoid and had irregular nuclei and scant cytoplasm. Mitotic activity was very low (up to 1 mitosis per 10 HPF). There was neither microvascular proliferation nor necrosis (Figure 3c). Altogether, the diagnosis is classified as CNS WHO Grade 2, IDH‐mutant astrocytoma, driven primarily by the histology illustrating low mitotic activity and the absence of higher‐grade features, together with the presence of an *IDH1* mutation, which was further supported by molecular findings.

After the patient’s subtotal resection, his neurological examination remained normal except for a mild right pronator drift. Additional surgery was deemed high risk, and the patient decided against further resection. Ultimately, the patient expressed a strong preference to defer radiotherapy and chemotherapy at the time but did agree with IDH‐inhibitor use. This approach is supported by data from the EORTC 22845 trial, which showed that delaying radiotherapy does not negatively affect overall survival, despite reduced progression‐free survival [18], and in light of the findings of the INDIGO trial [10], he proceeded with IDH‐inhibitor treatment. While vorasidenib was not yet FDA‐approved at the time of the patient’s resection, ivosidenib was available for off‐label use, per NCCN guidelines. The patient was therefore started on ivosidenib at a dose of 500 mg daily. However, after 4 months into his treatment, the T1 postcontrast MRI brain imaging demonstrated several new adjacent small foci of enhancement concerning for progression of disease (Figure 1c), and ivosidenib was discontinued at this point.

After thorough discussion with the patient and his various care teams, the decision was made to proceed with a second resection, which occurred 1 month later (5 months postinitiation of IDH inhibitor treatment). He underwent an awake redo of left craniotomy (Figure 1d) and had no complications or new neurologic deficits. The enhancing tumor was resected and sent for pathology analysis. Interestingly, the histology remained consistent with features of a Grade 2 astrocytoma: Histologic examination demonstrated that while the tumor now showed marked gemistocytic features and rare foci of foamy macrophages (Figure 3d), there was no evidence of mitotic activity, necrosis, or microvascular proliferation. Tumor cells stained positive for IDH1 R132H, and the Ki‐67 proliferative index remained low (2%–4%, Figure 3b). Sequencing redemonstrated mutations in *IDH1*, *ATRX*, and *TP53*. The copy number analysis plots, similarly, remained chromosomally stable (Figure 2b). There was also no *CDKN2A/B* deletion. The patient then received treatment comprised radiation therapy to the involved field receiving 60 Gy in 30 fractions followed by adjuvant temozolomide for 12 cycles. Eighteen months since the discovery of the patient’s tumor, his MRI brain continued to appear stable, and the patient has had normal neurologic examinations.

## 3. Discussion

IDH‐mutant Grade 2 astrocytomas have a relatively favorable prognosis when maximal safe resection is performed followed by standard therapies including radiation and temozolomide or observation [7–9]. The SOC now includes the option to use IDH inhibitors given they have shown promise in providing prolonged disease stabilization with a favorable toxicity profile [10, 16, 17, 19]. This case highlights radiographic challenges in the management of IDH‐mutant Grade 2 astrocytomas while undergoing treatment with IDH inhibitors and discordance of neuroradiological changes with histological and molecular findings.

For this case, the choice to start ivosidenib versus radiation and temozolomide postresection was driven by both the patient’s preference to defer and the promising efficacy of IDH inhibitors as a treatment in low‐grade gliomas [10, 16]. Ivosidenib was selected in this case given vorasidenib was not yet approved for its use. This represents a potential limitation, given vorasidenib has been demonstrated to have superior CNS penetration and has dual inhibition of both IDH1 and IDH2. Regardless, Mellinghoff et al. still demonstrated that both vorasidenib and ivosidenib significantly reduced tumor 2‐HG levels by greater than 90% [16]. Under RANO 2.0 criteria, this patient met radiographic criteria for progression based on new T1 postcontrast MRI after being on IDH inhibitor therapy for 4 months. While IDH inhibitors, including the newly FDA‐approved vorasidenib, have demonstrated efficacy in reducing 2‐HG levels and prolonging PFS in low‐grade astrocytomas [16], there remains limited data correlating new T1 postcontrast enhancement in the setting of IDH inhibitor therapy to pathology‐proven malignant transformation.

Although IDH inhibitors, such as vorasidenib, reduce 2‐HG and prolong PFS in Grade 2 IDH‐mutant gliomas [10, 16], published data directly linking new T1 postcontrast enhancement during therapy to pathology‐proven malignant transformation remain limited. Most published IDH‐inhibitor studies have, thus far, classified the astrocytomas as enhancing or nonenhancing at baseline and then assessed radiographic outcomes using RANO or RANO‐LGG to evaluate for stable disease or progression. Some studies performed volumetric analyses of nonenhancing T2/FLAIR tumor burden and tumor growth over time rather than correlating new enhancement with pathology seen on re‐resection [20, 21]. For example, in the Phase I ivosidenib study, stable disease was the best response in 30 of 35 patients (85.7%) with nonenhancing glioma, and exploratory volumetric analysis showed that the estimated tumor growth rate fell from 26% per six months before treatment to 9% per six months on therapy [20]. Similarly, early vorasidenib studies and the randomized perioperative vorasidenib and ivosidenib trial characterized radiographic response and biologic activity in resected tissue rather than correlating new enhancement with pathology [16, 21]. Additional studies with another IDH inhibitor, olutasidenib and DS‐1001, as well as a recent ivosidenib cohort, reported clinical activity, RANO‐based radiographic response, and in some cases on‐treatment tissue effects [17, 22, 23], but again with no imaging correlation with pathology when progression is noted.

Despite the new enhancement, on re‐resection, the tumor retained CNS WHO Grade 2 histologic findings of no necrosis or microvascular proliferation, nor increased proliferative activity. There were also no new molecular features to suggest progression, including no homozygous deletion of *CDKN2A/B*. These findings highlight that new contrast enhancement seen on MRI does not necessarily suggest malignant transformation, and changes seen on imaging may not always correlate with histologic or molecular progression. Therefore, in selected cases, a re‐resection may be helpful to clarify diagnosis and guide management when imaging may be discordant with the clinical and pathologic features. Overall, future studies that aim to evaluate tumor growth rate trajectories may help improve noninvasive response assessment [24].

The primary histologic change observed from the second resection was the emergence of gemistocytes. While the role of gemistocytes remains not well understood, they are traditionally considered relatively inert and nonproliferative [25]. However, some studies suggest that gemistocytes may arise from actively cycling tumor cells, potentially representing part of a more aggressive tumor phenotype [25–27]. While it does demonstrate an aggressive phenotype, its pathophysiology remains unknown, and importantly, it remains unclear whether this contributes to clinical behavior [28]. In contrast with our findings, at least one report has demonstrated a re‐resected tumor with a gemistocytic histologic phenotype, though notably with an associated increase in histologic grade [26]. Overall, further research is needed to clarify their biological significance and determine whether their presence may serve as a histologic marker of tumor aggressiveness [28, 29]. Another potential limitation is error in surgical sampling; however, the neurosurgeon was able to completely remove the enhancing region of recurrence in Figure 1d, making this possibility less likely.

In summary, there is a need for continued investigation of the role of novel genetic variants and molecular signatures to better predict tumor behavior. This patient case also highlights the importance of a multimodal assessment with integration of imaging, histology, and evolving molecular features to provide more personalized, effective treatment strategies.

## Funding

No funding was received for this manuscript.

## Disclosure

This case was previously presented in abstract form at the Society for Neuro‐Oncology Annual Meeting. The abstract was titled IMG‐90. Radiographic Enhancing Progression Without Molecular or Histologic Progression in IDH‐Mutant Grade 2 Astrocytoma Treated with an IDH Inhibitor: A Case Report [30].

## Conflicts of Interest

The authors declare no conflicts of interest.

## Data Availability

The data that support the findings of this study are available on request from the corresponding author. The data are not publicly available due to privacy or ethical restrictions.
